# Growth Description for Vessel Wall Adaptation: A Thick-Walled Mixture Model of Abdominal Aortic Aneurysm Evolution

**DOI:** 10.3390/ma10090994

**Published:** 2017-08-25

**Authors:** Andrii Grytsan, Thomas S. E. Eriksson, Paul N. Watton, T. Christian Gasser

**Affiliations:** 1 KTH Solid Mechanics, KTH Royal Institute of Technology, 100 44 Stockholm, Sweden; 2 Insigneo Institute for in silico Medicine, University of Sheffield, Sheffield S1 3JD, UK; a.grytsan@sheffield.ac.uk; 3 Swedish Defence Research Agency, 164 90 Stockholm, Sweden; thomas.eriksson@foi.se; 4 Department of Computer Science, University of Sheffield, Sheffield S1 4DP, UK; p.watton@sheffield.ac.uk; 5 Department of Mechanical Engineering and Materials Science, University of Pittsburgh, Pittsburgh, PA 15261, USA

**Keywords:** soft tissue, mixture model, growth, volume growth, vascular adaptation, G&R, AAA

## Abstract

(1) Background: Vascular tissue seems to adapt towards stable homeostatic mechanical conditions, however, failure of reaching homeostasis may result in pathologies. Current vascular tissue adaptation models use many *ad hoc* assumptions, the implications of which are far from being fully understood; (2) Methods: The present study investigates the plausibility of different growth kinematics in modeling Abdominal Aortic Aneurysm (AAA) evolution in time. A structurally motivated constitutive description for the vessel wall is coupled to multi-constituent tissue growth descriptions; Constituent deposition preserved either the constituent’s density or its volume, and Isotropic Volume Growth (IVG), in-Plane Volume Growth (PVG), in-Thickness Volume Growth (TVG) and No Volume Growth (NVG) describe the kinematics of the growing vessel wall. The sensitivity of key modeling parameters is explored, and predictions are assessed for their plausibility; (3) Results: AAA development based on TVG and NVG kinematics provided not only quantitatively, but also qualitatively different results compared to IVG and PVG kinematics. Specifically, for IVG and PVG kinematics, increasing collagen mass production accelerated AAA expansion which seems counterintuitive. In addition, TVG and NVG kinematics showed less sensitivity to the initial constituent volume fractions, than predictions based on IVG and PVG; (4) Conclusions: The choice of tissue growth kinematics is of crucial importance when modeling AAA growth. Much more interdisciplinary experimental work is required to develop and validate vascular tissue adaption models, before such models can be of any practical use.

## 1. Introduction

Soft tissue adaption (also called soft tissue growth and remodeling) is inherent to many biological processes and is driven by continuous turnover of the tissue’s cells and Extra Cellular Matrix (ECM) constituents. These mechanisms allows organs to not only change their shape, the alteration of tissue microstructure also enables changes to their (mechanical) properties. Most interestingly, external mechanical stimuli influence cell function at the level of gene expression and thereby contribute to the overall control of soft tissue adaptation. Soft biological tissues seem to adapt towards stable homeostatic mechanical conditions and failure of reaching homeostasis may result in pathologies. For example, tumor growth, atherosclerosis, aneurysm disease, dilated cardiomyopathy and fibrosis are examples of pathologies associated with maladaptive soft tissue adaption. Models capable of predicting tissue adaption would not only be of considerable scientific interest, but would also have a large number of practical applications. Consequently, the mechanics-related aspects of soft tissue adaptation have attracted increasing research attention and comprehensive review articles are available in the literature [1,2,3,4,5,6,7].

The biomechanical characteristics of the passive vascular wall are predominantly determined by the ECM structural proteins collagen and elastin. Collagen provides the tissue with stiffness, strength and toughness, and is continuously turned over with a typical half-life in the range of several weeks to months. The maintenance of the collagen structure relies on a delicate (coupled) balance between degradation, mainly through Matrix MetalloProteinases (MMPs), and synthesis by cells like Smooth Muscle Cells (SMC), fibroblasts and myofibroblasts [8]. Such cells are anchored to collagen fibers and respond to mechanical strain or stress by adjusting their expression and synthesis of collagen molecules [9,10]. Mechanical stimulus not only promotes collagen synthesis [11], it also provides protection from collagen degradation [12,13]. Elastin functions in partnership with collagen, and endows vascular tissue with elasticity. Elastin synthesis normally ceases soon after puberty once the body reaches maturity. While elastin is extremely insoluble and stable with half-life times in the order of tens of years [14], it may be degraded by selective MMPs (collectively called elastase), a mechanism important for many physiological processes like elastogenesis and repair [15].

Vascular tissue is at a state of continuous mass turnover, which causes growth and remodeling at a local tissue level. Specifically, growth changes the tissue’s stress-free configuration, while remodeling alters the tissue mechanical properties through modifying its internal structure. The Kinematics-based Adaptation (KA) and the Constrained Mixture Model-based Adaptation (CMMA) are the two most commonly used frameworks to model such phenomena.

Similar to elasto-plastic kinematics [16], KA hypothesizes that the adapting vascular tissue can be described by multiplicative decomposition of the total deformation [17,18]. Here, the deformation gradient $F^{g}\left(\tau \right)$ entirely specifies growth kinematics by introducing a stress-free intermediate configuration, with respect to which the deformation gradient $F^{e}\left(t\right)$ records all non-growth-related deformations. The two distinct time scales, τ and *t*, are related to tissue growth (mass turnover) and external mechanical loading, respectively. The tissue can be regarded as a single-phase continuum or as a multi-phase mixture of tissue constituents. Pioneering models of the soft tissue growth related the local stress or strain states to the volume change of the tissue [18,19], an approach that has been frequently pursued in the literature [20,21].

While KA gives physically reasonable results, it fails to explicitly describe the continuous production and degradation of vascular tissue constituents. In contrast, CMMA focuses on stress-mediated mass production and removal, not kinematics. Each relevant tissue constituent is considered to be in continuous turnover at a specific density rate, i.e., mass per current volume per time. Such an approach *a priori* considers different constituents, like elastin and collagen, that turnover at different rates in the vessel wall. To date, several versions and applications of this idea have been reported in the literature [22,23,24]. CMMA has been linked with the theory of volumetric growth [18] in order to cope with 3D problems [25], additionally to the microstructural description of collagen fiber orientation [26]. Most interestingly, the recently developed homogenized CMMA [27] used KA kinematics concept and reported very similar predictions than the original CMMA. This key observation indicates that the simpler KA is indeed able to capture the overall consequences of the continuous production and degradation of tissue constituents.

In order to close a 3D adaption model, both theories KA and CMMA require to specify how tissue volume change is turned into growth deformation, i.e., how a scalar quantity maps into a second-order tensor. Due to lack of experimental information, this step requires making assumptions about the underlying growth kinematics. Most commonly, isotropic volumetric growth is assumed [3,28,29,30], probably because of its simplicity and rather straightforward implementation. However, unrealistic model predictions have been reported when assuming isotropic volume growth [29].

Blood vessels are hollow organs and most collagen fibers in the vessel wall are distributed tangentially around the lumen [31,32,33,34,35]. Consequently, newly synthesized collagen is clearly not isotropically added to the vessel wall, which in turn motivates anisotropic growth kinematics to describe the adapting vessel wall. Anisotropic growth kinematics are uniquely determined by eigenvalues and eigenvectors of the growth-related deformation, and the literature reports different such particularizations. Approaches propose transverse isotropic [36,37], general orthotropic [38] and principal stress-driven [20] growth kinematics. Most importantly, the predictions of a recent arterial remodeling study [39] reported high sensitivity with respect to the selected growth kinematics.

In conclusion, the scarcity of relevant experiment data restrains from a sound understanding of vascular tissue adaption. While experimental studies remain technically challenging, biomechanical modeling may help testing different mechanics-dominated hypotheses of vascular tissue adaption. The present work used biomechanics simulation to explore the influence of different growth kinematics on the outcome of Abdominal Aortic Aneurysm (AAA) growth predictions. AAA is a cardiovascular pathology characterized by the localized enlargement of the aorta at its infrarenal segment. Significant enlargement may lead to subsequent rupture, which can have major consequences for the patient, causing death or severe disability. AAA growth is not homogenous [40,41] and depends on many factors [42,43,44], such that predicting AAA development in individual patients could usefully complement clinical decision making. In addition, a reliable AAA growth model may help to explore the interaction of tissue growth mechanisms towards designing therapeutic drugs that could stabilize AAA growth. For the present study we further developed a previously proposed constitutive model for volumetric growth [30,45], enabling anisotropic growth kinematics and systematically explored AAA growth predictions with respect to prescribed growth kinematics as well as some baseline vessel wall properties.

## 2. Methods

The artery is modeled as a cylindrical structure formed by medial and adventitial layers. The media is composed by elastin, ground matrix and two helically arranged families of collagen fibers. The adventitia is composed by ground matrix and another two helically arranged families of collagen fibers. The ground matrix collectively represents the mechanical properties of passive SMC, and all ECM constituents other than elastin and collagen. The time is separated into a short time scale *t* of seconds, and a long time scale, τ of weeks, related to the cardiac cycle time and the time needed for vascular tissue adaption, respectively. At the time scale *t* the tissue is considered quasi-incompressible.

### 2.1. Mixture Theory

Vascular tissue is modeled as a constrained mixture at affine deformation. Consequently, all tissue constituents (ground matrix, elastin and collagen) are simultaneously present at a material point and follow the deformation of the continuum. In addition, the sum of changes in individual constituent’s mass and volume sum up to the total change of tissue mass and volume.

**Figure 1:**
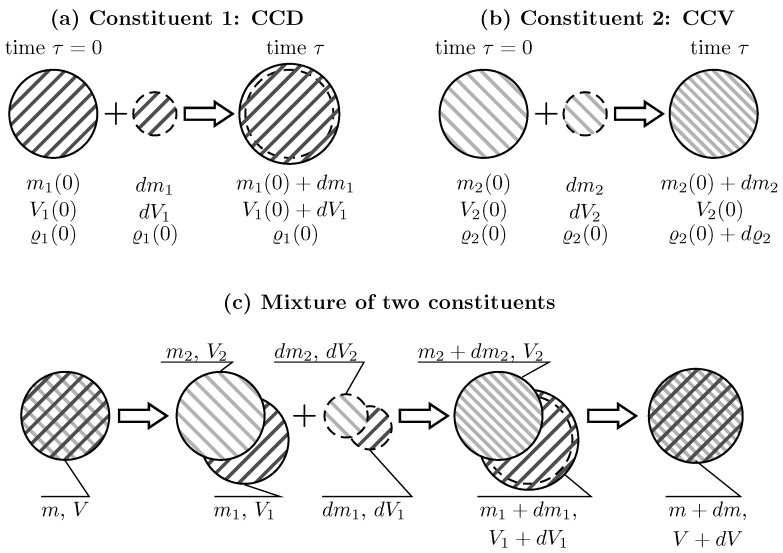
Illustration of different scenarios for adding mass to individual constituents and a mixture of two constituents. (**a**) Constituent 1, constant density (CCD): adding the mass $dm_{1}$ of the volume $dV_{1}$ to the initial mass $m_{1}\left(0\right)$ of the volume $V_{1}\left(0\right)$ results in increased mass $m_{1}+dm_{1}$ and volume $V_{1}+dV_{1}$. However, the density $\varrho_{1}\left(\tau \right)=\varrho_{1}\left(0\right)$ remains constant; (**b**) Constituent 2, constant volume (CCV): adding the mass $dm_{2}$ of the density $\varrho_{2}\left(\tau \right)=\varrho_{2}\left(0\right)$ to the initial mass $m_{2}\left(0\right)$ of density $\varrho_{2}\left(0\right)$ results in increased mass $m_{2}+dm_{2}$ but unchanged volume $V_{2}\left(\tau \right)=V_{2}\left(0\right)$. Hence, the density $\varrho_{2}\left(\tau \right)=\varrho_{2}\left(0\right)+d\varrho_{2}$ increases; (**c**) Mixture of Constituent 1 (following CCD) and Constituent 2 (following CCV): The tissue of mass *m* and volume *V* is composed of Constituent 1 ($m_{1},\;V_{1}$) and Constituent 2 ($m_{2},\;V_{2}$). The mass increments $dm_{1},\;dm_{2}$ of volumes $dV_{1},\;dV_{2}$ are added to each constituent. This results in increased masses of both constituents, increased volume of Constituent 1 and unchanged volume of Constituent 2. Over all the mass m+dm and the volume V+dV of the mixture have been changed.

Due to the lack of experimental evidence, the kinematics by which tissue constituents are added and removed from arterial tissue are unclear. In the present study, two extremal cases are investigated, Constant Constituent Density (CCD) and Constant Constituent Volume (CCV) [30], respectively. CCD adds/removes constituent’s mass without changing its density, whereas CCV adds/removes constituent’s mass without changing its volume, see Figure 1a,b.

#### 2.1.1. Deposition at Constant Constituent Density (CCD)

Figure 1a illustrates CCD-based deposition of constituent mass. At time τ=0, Constituent 1 (collagen for example) has mass $m_{1}\left(0\right)$, volume $V_{1}\left(0\right)$ and density $\varrho_{1}\left(0\right)$. Subsequently, at time τ, a mass $dm_{1}$ of volume $dV_{1}$ and density $\varrho_{1}\left(0\right)$ is added. As a result, the mass and the volume at the current time τ increase to $m_{1}\left(\tau \right)=m_{1}\left(0\right)+dm_{1}$ and $V_{1}\left(\tau \right)=V_{1}\left(0\right)+dV_{1}$, respectively. In contrast, the density remains constant at $\varrho_{1}\left(0\right)=\varrho_{1}\left(\tau \right)$. CCD-based deposition defines the normalized changes of volume ${\hat{v}}_{1}=V_{1}\left(\tau \right)/V_{1}\left(0\right)$, mass ${\hat{m}}_{1}=m_{1}\left(\tau \right)/m_{1}\left(0\right)$ and density ${\hat{\varrho }}_{1}=\varrho_{1}\left(\tau \right)/\varrho_{1}\left(0\right)\equiv 1$.

#### 2.1.2. Deposition at Constant Constituent Volume (CCV)

Figure 1b illustrates CCV-based deposition of constituent mass. Constituent 2 (elastin for example) with mass $m_{2}\left(0\right)$, volume $V_{2}\left(0\right)$ and density $\varrho_{2}\left(0\right)$ accommodates the additional mass $dm_{2}$ of the same density $\varrho_{2}\left(0\right)$. As a result, the mass increases to $m_{2}\left(\tau \right)=m_{2}\left(0\right)+dm_{2}$ while the volume $V_{2}\left(\tau \right)=V_{2}\left(0\right)$ remains constant. This leads to increased constituent density $\varrho_{2}\left(\tau \right)=\varrho_{2}\left(0\right)+d\varrho_{2}$. Hence for CCV-based deposition, the normalized changes of mass and density are ${\hat{m}}_{2}=m_{2}\left(\tau \right)/m_{2}\left(0\right)$ and ${\hat{\varrho }}_{2}=\varrho_{2}\left(\tau \right)/\varrho_{2}\left(0\right)$, respectively. Given that the volume remains constant, the normalised volume ${\hat{v}}_{2}=V_{2}\left(\tau \right)/V_{2}\left(0\right)\equiv 1$,

#### 2.1.3. Mixed Deposition

Different constituents may be differently integrated in the vascular wall. For example, collagen may follow CCD-based deposition, and elastin may follow CCV-based deposition. This is illustrated in Figure 1c. Here, two constituents ζ=1,2 are considered, with Constituent 1 being CCD-based and Constituent 2 being CCV-based deposited. At time τ=0, the tissue has mass m(0), volume V(0) and density ρ(0). It is composed of two constituents, each of which has mass $m_{\zeta }\left(0\right)$, volume $V_{\zeta }\left(0\right)$ and initial volume fractions $\phi_{\zeta }\left(0\right)=V_{\zeta }\left(0\right)/V\left(0\right)$. In addition to the constituent densities $\varrho_{\zeta }\left(0\right)$, partial densities $\rho_{\zeta }\left(0\right)=m_{\zeta }\left(0\right)/V\left(0\right)$ are defined with respect to the tissue’s volume V(0). Then, the mass $dm_{\zeta }$ with the volume $dV_{\zeta }$ and the density $\varrho_{\zeta }\left(0\right)$ is added to each constituent. At time τ, the mass, volume, and density of the individual constituents are changed as described above, while the change of partial densities of constituents is ${\hat{\rho }}_{\zeta }\left(\tau \right)=\rho_{\zeta }\left(\tau \right)/\rho_{\zeta }\left(0\right)$.

The tissue’s volume change is now computed using the volume changes of constituents and their initial volume fractions, $\hat{v}\left(\tau \right)=\sum_{\zeta =1}^{N}{\hat{v}}_{\zeta }\left(\tau \right)\phi_{\zeta }\left(0\right)$. Taking into account that $\hat{v}\left(\tau \right)\equiv {\hat{m}}_{\zeta }\left(\tau \right)$ holds for a constituent following CCD, while $\hat{v}\left(\tau \right)\equiv 1$ holds for a constituent following CCV, the normalized change of volume of a multi-constituent tissue is given as

(1) $$\hat{v}\left(\tau \right)=\sum_{\zeta =1}^{N}{\hat{v}}_{\zeta }\left(\tau \right)\phi_{\zeta }\left(0\right)=\sum_{\zeta \in \mathrm{CCD}}{\hat{m}}_{\zeta }\left(\tau \right)\phi_{\zeta }\left(0\right)+\sum_{\zeta \in \mathrm{CCV}}\phi_{\zeta }\left(0\right).$$

Such defined normalized tissue volume change $\hat{v}$ specifies the growth kinematics according to Section 2.2. Likewise, the normalized mass changes ${\hat{m}}_{\zeta }$ and the partial density changes ${\hat{\rho }}_{\zeta }$ of individual constituents ζ are used to connect tissue adaptation to its elastic response.

### 2.2. Kinematics

**Figure 2:**
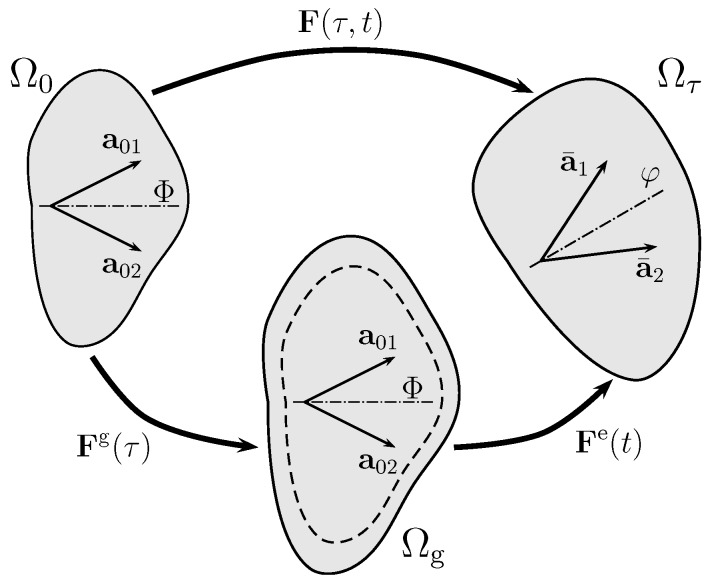
Kinematics of growth. Deformation gradient F(τ,t) maps reference configuration $\Omega_{0}$ to the current configuration $\Omega_{\tau }$. The growth tensor $F^{g}\left(\tau \right)$ (with $\det F^{g}=\det F=\hat{v}$) connects reference configuration $\Omega_{0}$ to the intermediate stress-free configuration $\Omega_{g}$, which accounts for the volume change at the long time-scale τ. Then, the elastic deformation tensor $F^{e}\left(t\right)$ connects $\Omega_{g}$ to the current configuration $\Omega_{\tau }$. That is, the total deformation gradient F(τ) is split into volumetric growth part $F^{g}$ and elastic part $F^{e}$.

Adaptation-related and elastic deformations of the artery are associated with different time scales denoted τ and *t*, respectively. As shown in Figure 2, the two time scales can be separated by the multiplicative decomposition of the deformation gradient $F\left(\tau ,t\right)=F^{e}\left(t\right)F^{g}\left(\tau \right)$, where the volumetric growth tensor $F^{g}\left(\tau \right)$ accommodates the change of volume ($\det F^{g}=\det F=\hat{v}\left(\tau \right)$), while the elastic deformation gradient $F^{e}\left(t\right)$ is restricted to nearly incompressible elastic deformation, i.e., $\det F^{e}\to 1$. 

The tissue volume change $\hat{v}\left(\tau \right)$ is given by the sum of the volume changes of all tissue constituents according to Equation (1). Due to limited experimental data regarding the kinematics of vascular tissue adaption, idealized growth kinematics are introduced in Section 2.2.3.

#### 2.2.1. Quasi-Incompressible Elastic Response

The elastic deformation gradient $F^{e}\left(t\right)$ is further split into volumetric and isochoric parts, $F^{e}\left(t\right)={\left(J^{e}\right)}^{1/3}\bar{F}$ with $J^{e}=\det F^{e}$ and $\det\bar{F}=1$. According to the volumetric-isochoric split, the elastic right and left Cauchy-Green strains are $C^{e}=F^{\mathrm{eT}}F^{e}={\left(J^{e}\right)}^{2/3}\bar{C}$ and $b^{e}=F^{e}F^{\mathrm{eT}}={\left(J^{e}\right)}^{2/3}\bar{b}$, with the three strain invariants ${\bar{I}}_{1}=\mathrm{tr}\bar{C}=\mathrm{tr}\bar{b}$, ${\bar{I}}_{2}=\mathrm{tr}{\bar{C}}^{-1}=\mathrm{tr}{\bar{b}}^{-1}$ and ${\bar{I}}_{3}=\det\bar{C}\equiv 1$. In order to achieve an incompressible elastic deformation, the bulk modulus of the vessel wall tissue becomes a penalty parameter, such that $J^{e}\to 1$.

#### 2.2.2. Collagen Structure and Reference Configuration

As illustrated in Figure 2, collagen is assumed to be arranged into two fiber families of referential directions $a_{0i}$, i=1,2. For brevity, the index *i* is neglected in the following. The referential collagen structure is represented by a structural tensor $A_{0}=a_{0}⊗a_{0}$ with the symbol ⊗ denoting the dyadic vector product. This allows for the introduction of an additional isochoric invariant ${\bar{I}}_{4}=\bar{C}:A_{0}={\bar{\lambda }}_{0}^{2}$, where ${\bar{\lambda }}_{0}$ yields the isochoric tissue stretch along the reference direction $a_{0}$. Finally, with the isochoric spatial fiber direction $\bar{a}=\bar{F}a_{0}$, the spatial collagen structure is represented by the spatial structural tensor $\bar{A}=\bar{a}⊗\bar{a}=\bar{F}a_{0}⊗\bar{F}a_{0}=\bar{C}:\left(a_{0}⊗a_{0}\right)=\bar{C}:A_{0}$.

The natural reference configurations of collagen fibers and the surrounding tissue may be different, and this mismatch is represented by an isochoric recruitment stretch variable ${\bar{\lambda }}_{r}>0$ [46]. This variable determines the tissue stretch in the direction $\bar{a}$, at which the collagen fiber starts to bear load, i.e., starts to store elastic energy. In addition, collagen fibers are thought to have neither compressive nor bending stiffness, such that with ${\bar{\lambda }}_{c}^{2}={\bar{I}}_{4c}$ and ${\bar{I}}_{4r}={\bar{\lambda }}_{r}^{2}$, the square of collagen fiber stretch

(2) $${\bar{I}}_{4c}=\max\left[\frac{{\bar{I}}_{4}}{{\bar{I}}_{4r}},\;1\right]\;$$

is sufficient to represent the fibers’s strain state. Recruitment stretch ${\bar{\lambda }}_{r}$ adaption in response to alterations of strain variables is detailed in Section 2.3.2.

#### 2.2.3. Growth Tensor description

With the two collagen fiber families spanning the tangential plane (see Figure 2) with the unit normal $n=a_{01}\times a_{02}$, the general form of transversely anisotropic growth yields

(3) $$F^{g}=\alpha I+\beta n⊗n\;$$

where I denotes the second order identity tensor. The parameters α and β control growth kinematics and are constrained by the normalized volume change ($\hat{v}$) such that $\det F^{g}=\hat{v}$ holds.

The case of Isotropic Volume Growth (IVG) is obtained through $\alpha ={\hat{v}}^{1/3}$, β=0 and thus

(4) $$F^{g}={\hat{v}}^{1/3}I\;$$

yields IVG-growth deformation. Alternatively, setting $\alpha ={\hat{v}}^{1/2}$ and $\beta =1-{\hat{v}}^{1/2}$ we have the case of in-Plane Volume Growth (PVG), where new volume is deposited only within the tangential plane. Hence

(5) $$F^{g}={\hat{v}}^{1/2}I+\left(1-{\hat{v}}^{1/2}\right)n⊗n\;,$$

gives PVG-growth deformation.

Lastly, specifying α=1, $\beta =\hat{v}-1$ gives the special case of in-Thickness Volume Growth (TVG), where new volume is deposited perpendicular to the tangential plane and thus

(6) $$F^{g}=I+\left(\hat{v}-1\right)n⊗n.$$

describes TVG-growth deformation.

#### 2.2.4. Implication of Growth Kinematics on the Equi-Biaxial Tissue Properties

In order to explore how different growth kinematics may influence the elastic properties of the vessel wall, we consider a vessel wall patch of referential dimensions L×L×H subject to equi-biaxial deformation. For this example, the total deformation reads $F=\mathrm{diag}\left[\lambda ,\lambda ,\lambda_{3}\right]$, and $F^{e}=F{\left(F^{g}\right)}^{-1}$ defines the elastic deformation of the vessel wall patch. Using the growth kinematics detailed in Section 2.2.3, i.e., Equations (4)–(6), the elastic deformations yield

(7) $$\begin{array}{c} F^{e}={\hat{v}}^{-1/3}\mathrm{diag}\left[\lambda ,\lambda ,\lambda_{3}\right]\;,\;F^{e}=\mathrm{diag}\left[{\hat{v}}^{-1/2}\lambda ,{\hat{v}}^{-1/2}\lambda ,\lambda_{3}\right]\;,\;F^{e}=\mathrm{diag}\left[\lambda ,\lambda ,{\hat{v}}^{-1}\lambda_{3}\right]\;,\; \end{array}$$

for IVG, PVG and TVG kinematics, respectively.

In addition to the kinematics, the tissue’s constitution needs to be specified. Here, for simplicity, the vessel wall is considered to be a neo-Hookean material. Consequently, the Cauchy stress reads

(8) $$\sigma =-p_{h}I+\hat{\rho }\mu C^{e},$$

with μ being the tissue’s referential stiffness, and $p_{h}$ denoting the hydrostatic pressure to enforce incompressibility. Due to mechanical equilibrium, the force $F_{0}$ acting on the tissue patch determines the Cauchy $\sigma =F_{0}/\left(\lambda L\lambda_{3}H\right)\left(I-e_{3}⊗e_{3}\right)$, where $e_{3}$ denotes the out-of-plane direction. Finally, Equation (8) can be given for different growth models, and the equibiaxial stretch λ can be derived from

(9) $$\begin{array}{c} P=\hat{\rho }{\hat{v}}^{1/3}\mu \left(\lambda -{\hat{v}}^{2}\lambda^{-5}\right)\;,\;P=\hat{\rho }\mu \left(\lambda -{\hat{v}}^{3}\lambda^{-5}\right)\;,\;P=\hat{\rho }\hat{v}\mu \left(\lambda -\lambda^{-5}\right)=\hat{m}\mu \left(\lambda -\lambda^{-5}\right)\;,\; \end{array}$$

for IVG, PVG and TVG kinematics, respectively. Here, $P=F_{0}/LH$ denotes the first Piola-Kirchhoff stress in the wall, and it is worth noting that for TVG, the product of the normalized changes of the density $\hat{\rho }$ and the volume $\hat{v}$ yield the normalized mass change $\hat{m}=\hat{\rho }\hat{v}$.

**Figure 3:**
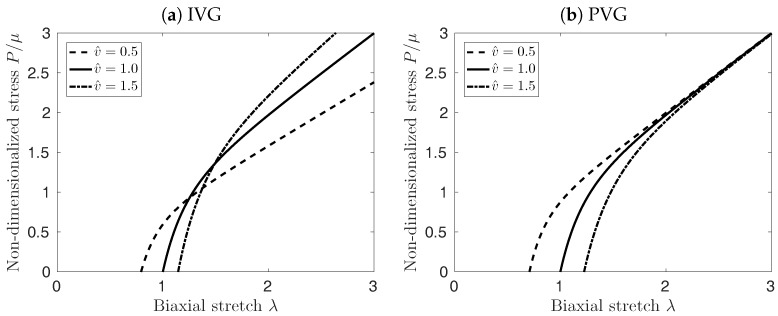

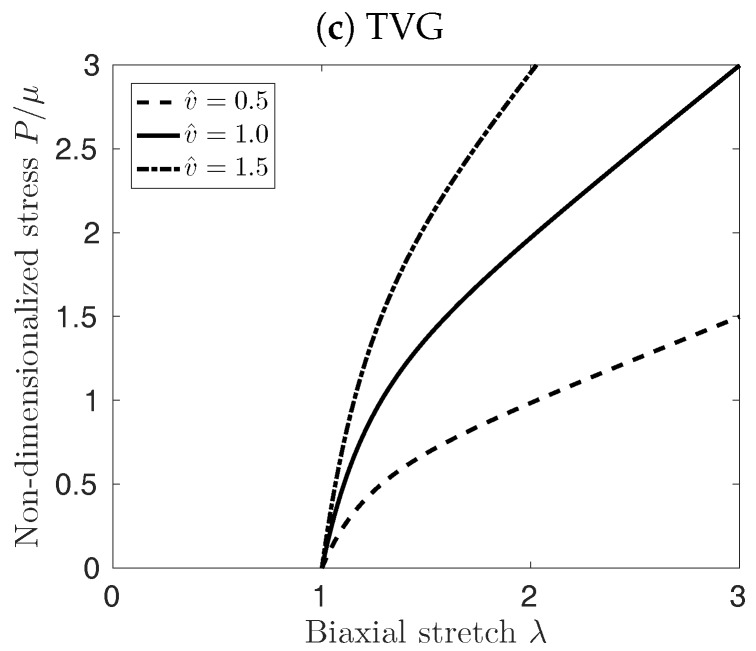
Effect of Isotropic Volume Growth (IVG), in-Plane Volume Growth (PVG), and in-Thickness Volume Growth (TVG) kinematics on the stress-stretch properties of the neo-Hookean material under equi-biaxial extension. First Piola-Kirchoff Stress (*P*) versus stretch (λ) is depicted for constant $\hat{v}=1.0$ (solid curves), decreasing $\hat{v}=0.5$ (dashed curves), and increasing $\hat{v}=1.5$ (dash-dotted curves) tissue volumes.

Figure 3 illustrates the implication of the different cases for growth kinematics on the mechanical properties of the vessel patch under equi-biaxial extension. PVG affects the edge length of the patch in stress-free configuration, which in turn affects mostly the material response at biaxial stretches smaller than λ=2. At large stretches λ, the material response is not influenced by the volume change, see Figure 3b. Conversely, TVG does not affect the edge length of the patch, and only the slope of the curves in Figure 3c is affected by the volume change. Finally, IVG exhibits mixed behavior, modifying both, the stress-free edge length of the patch as well as the slope of the curves for large λ, see Figure 3a.

### 2.3. Constitutive Modeling of Vascular Tissue

#### 2.3.1. Elastic Properties

Vascular tissue is modeled as a quasi-incompressible material and an additive split of the strain-energy function

(10) $$\Psi =U\left(J^{e}\left(t\right)\right)+\bar{\Psi }\left({\bar{I}}_{1},{\bar{I}}_{4ci}\right),$$

into its volumetric and isochoric parts is followed. Here, $U\left(J^{e}\left(t\right)\right)=\mu_{\kappa }{\left[J^{e}\left(t\right)-1\right]}^{2}/2$ is a penalty energy that enforces quasi-incompressibility, where the artificial bulk modulus $\mu_{\kappa }$ serves as penalty parameter.

The isochoric strain energy $\bar{\Psi }$ is modeled as the sum of the strain energies of the individual constituents, i.e.,

(11) $$\bar{\Psi }={\hat{\rho }}_{e}{\bar{\Psi }}_{e}\left({\bar{I}}_{1}\right)+{\hat{\rho }}_{g}{\bar{\Psi }}_{g}\left({\bar{I}}_{1}\right)+\sum_{i=1,2}{\hat{\rho }}_{ci}{\bar{\Psi }}_{ci}\left({\bar{I}}_{4ci}\right)\;$$

where ${\bar{\Psi }}_{e},{\bar{\Psi }}_{g}$ and ${\bar{\Psi }}_{ci}$ denote the strain energies of elastin, ground matrix and the i–th family of collagen fibers, and ${\hat{\rho }}_{e},{\hat{\rho }}_{g}$ and ${\hat{\rho }}_{ci}$ are the corresponding normalized partial densities. These normalized densities are specified by the growth of the individual vessel wall constituents according to Section 2.1. Due to negligible amount of elastin in the adventitia, we set ${\hat{\rho }}_{e}=0$ in this vessel layer.

The neo-Hookean strain energy

(12) $${\bar{\Psi }}_{\zeta }=\frac{\mu_{\zeta }}{2}\left({\bar{I}}_{1}-3\right)\;,\;\zeta =e,g$$

is used to capture the isotropic responses of elastin and ground substance, where $\mu_{e}$ and $\mu_{g}$ denote their referential stiffnesses, respectively.

Following [46], the elastic response of the i–the family of collagen fibers is modeled by an exponential strain energy [47] relative to the configuration the fibres are recruited to load bearing

(13) $${\bar{\Psi }}_{ci}=\frac{k_{1}}{2k_{2}}\left\{\exp\left[k_{2}{\left({\bar{I}}_{4ci}-1\right)}^{2}\right]-1\right\},\;i=1,2,$$

where $I_{4c}$ denotes the square of the collagen fibre stretch (see Equation (2)) and $k_{1},k_{2}$ are the material parameters.

The second Piola-Kirchhoff tensor $S^{e}=2\partial \Psi /\partial C^{e}$ is defined at the intermediate configuration $\Omega_{g}$, and reads with the use of the strain energy Equation (11) and the chain rule,

(14) $$S^{e}=J^{e}p_{h}{\left(C^{e}\right)}^{-1}+2{\left(J^{e}\right)}^{-2/3}\left\{\left[{\hat{\rho }}_{e}{\bar{\Psi }}_{e,1}+{\hat{\rho }}_{g}{\bar{\Psi }}_{g,1}\right]\mathrm{Dev}\left(I\right)+\sum_{i=1,2}{\hat{\rho }}_{ci}{\bar{\Psi }}_{ci,4}\mathrm{Dev}\left(A_{0,i}\right)\right\},$$

where $\mathrm{Dev}\left(•\right)=\left(•\right)-\left(1/3\right)\left[\left(•\right):C^{e}\right]{\left(C^{e}\right)}^{-1}$ is the material deviatoric operator, and $p_{h}=\partial U/\partial J^{e}=\mu_{\kappa }\left(J^{e}-1\right)$. Equation (14) introduced the stress factors

(15) $${\bar{\Psi }}_{\zeta ,1}=\frac{\partial {\bar{\Psi }}_{\zeta }}{\partial {\bar{I}}_{1}}=\frac{\mu_{\zeta }}{2}\;,\zeta =e,g\;\;;\;\;{\bar{\Psi }}_{ci,4}=\frac{\partial {\bar{\Psi }}_{ci}}{\partial {\bar{I}}_{4i}}=k_{1}{\bar{I}}_{4ri}^{-1}\left({\bar{I}}_{4ci}-1\right)\exp\left[k_{2}{\left({\bar{I}}_{4ci}-1\right)}^{2}\right]\;,$$

defined through the partial derivatives of Equations (12) and (13), respectively.

Finally, the Cauchy stress of the vessel wall is defined by a push-forward operation of the second Piola-Kirchhoff tensor (14), i.e., $\sigma ={\left(J^{e}\right)}^{-1}F^{e}S^{e}F^{\mathrm{eT}}$. Thus,

(16) $$\sigma =p_{h}I+2{\left(J^{e}\right)}^{-1}\left\{\left[{\hat{\rho }}_{e}{\bar{\Psi }}_{e,1}+{\hat{\rho }}_{g}{\bar{\Psi }}_{g,1}\right]\mathrm{dev}\left(\bar{b}\right)+\sum_{i=1,2}{\hat{\rho }}_{ci}{\bar{\Psi }}_{ci,4}\mathrm{dev}\left({\bar{A}}_{i}\right)\right\},$$

where $\mathrm{dev}\left(•\right)=\left(•\right)-\left(1/3\right)\left[(•):I\right]I$ denotes the spatial deviator operator.

#### 2.3.2. Collagen Turnover Modeling

The collagen fabric of arterial walls is continuously remodeled through cell-based synthesis and MMP-based degradation [8], at a half-life time of approximately two months. In order to maintain the structural integrity of the vessel wall, newly formed collagen fibers are deposited (or integrated) at a certain attachment stretch ${\bar{\lambda }}_{a}$, a quantity also denoted as deposition stretch or pre-stretch [46]. The stretch difference between collagen that is already in the vessel wall ${\bar{\lambda }}_{c}$ and collagen that is deposited ${\bar{\lambda }}_{a}$, is thought to be sensed by cells and stimulates collagen turnover. Specifically, the state of collagen is thought to develop in response to the stretch-based stimulus

(17) $$\xi =\frac{{\bar{I}}_{4c}-{\bar{I}}_{4a}}{{\bar{I}}_{4a}-1}$$

with ${\bar{I}}_{4a}={\bar{\lambda }}_{a}^{2}$ and ${\bar{I}}_{4c}={\bar{\lambda }}_{c}^{2}$. At homeostasis, collagen is deposited at the same stretch as collagen that is already in the vessel wall, i.e., ${\bar{I}}_{4c}-{\bar{I}}_{4a}=0$, and the stimulus tends to zero ξ→0.

The stretch-based stimulus ξ determines the development of collagen’s recruitment stretch ${\bar{\lambda }}_{r}$ as well as its normalized mass ${\hat{m}}_{c}$. Specifically, these state variables change with respect to the remodeling time scale τ, and follow the rate equations

(18) $$\frac{\partial {\bar{\lambda }}_{r}}{\partial \tau }=\alpha \xi \;\;;\;\;\frac{\partial {\hat{m}}_{c}}{\partial \tau }=\beta {\hat{m}}_{c}\xi \;,$$

were, α and β are rate constants related to changes of collagen recruitment stretch and mass, respectively. The material parameters $k_{1},k_{2}$ of collagen, as well as the collagen fiber orientation $a_{0}$ are kept constant over time.

#### 2.3.3. Elastin Degradation

Aneurysm development is associated with up to 90% loss of elastin [48], the exact cause of which is not fully understood. While the initial elastin degradation might be caused by non-physiological hemodynamic conditions like low levels of Wall Shear Stress (WSS), or any type of vessel wall injury, degradation may be accelerated by the existence of Intra-Luminal Thrombus (ILT). The ILT is a blood clot that forms inside most AAAs [49]. The different causes and mechanisms of elastin degradation are likely coupled. For simplicity, such coupling effects are neglected, and the isolated effect of growth kinematics on AAA expansion is investigated in this work. To this end, elastin degradation is prescribed by a simple exponential decay function ${\hat{m}}_{e}={\hat{m}}_{e}\left(X,\tau \right)$, see Section 2.4.

### 2.4. Axisymmetric Aorta Model

A cylindrical infrarenal aorta segment of length *L* is used to predict AAA onset and progression. Axisymmetry as well as distal-proximal symmetry are assumed, and one-eighth of the infrarenal aorta segment was modeled. The segment is composed of medial and adventitial layers, and the finite element mesh consists of 1920 elements: 30 elements in axial direction, 8 in circumferential direction, and 4 through the thickness of each vessel wall layer. The simulation workflow is divided into two phases.

In the first phase, the infrarenal aorta is exposed to a fixed axial pre-stretch $\lambda_{z}$ and inflated at an internal pressure $p_{i}$. Then collagen turns over at fixed mass until homeostatic conditions are reached, i.e., the recruitment stretch develops according to Equation (18)${}_{1}$ until $\lambda_{c}$ is spatially uniform and equal to the attachment stretch $\lambda_{a}$.

In the second phase, boundary and loading conditions remain unchanged, remodeling time is set to τ=0, and the aneurysm starts to develop. To this end, elastin mass is prescribed by

(19) $${\hat{m}}_{e}\left(z,\tau \right)=1-\left(1-c_{\min}^{\tau /T}\right)\exp\left[-m_{1}{\left(2z/L-1\right)}^{2}\right]\;,$$

where *z* is the axial Lagrangian coordinate. Specifically, elastin degradation is prescribed such that the amount $c_{\min}$ of elastin remains left after the time τ=T in the center of the vessel, i.e., at z=L/2. In addition, the elastin degradation profile is controlled by the parameter $m_{1}$, and such defined profile has been previously used [46].

In response to altered elastin mass, collagen adapts following Equations (18), and Equation (1) defines the corresponding normalized volume changes. Finally, displacement and strain are computed by solving the quasi-static Cauchy equation of motion divσ=0 with div(•) denoting the divergence operator. Then, the time is incremented τ⇐τ+Δτ and the procedure repeated.

### 2.5. Parameter Study

The parameter study investigates the sensitivity of model predictions with respect to key model parameters. Predictions are compared to baseline results that are obtained with model parameters listed in Table 1. These parameters have either been used previously in the literature, and/or they were set to plausible values. In addition to the growth kinematics described in Section 2.2.3, i.e., IVG, PVG, and TVG, an isochoric case, denoted as No Volume Growth (NVG), was used to fully explore AAA growth results. For NVG, elastin and collagen depositions followed CCV mixture model, introduced in Section 2.1. For the other growth kinematics, elastin or collagen followed the study-dependent mixture models detailed below.

#### 2.5.1. Study 1: Influence of Collagen Net Growth

Collagen and elastin deposition followed CCD and CCV mixture model, respectively. Model sensitivity to the collagen net growth parameter β={0.75,1.00,1.25} (year^−1^) is tested and compared to baseline prediction.

#### 2.5.2. Study 2: Influence of Initial Volume Fractions

For the different growth kinematics, the influence of the initial volume fractions of elastin and collagen is investigated. The deposition of questioned constituent (elastin or collagen) followed CCD mixture model, while others followed CCV. Specifically, model sensitivity to the initial volume fractions of elastin of $\phi_{e}=\left\{0.12,0.18\right\}$ and collagen of $\phi_{c}=\left\{0.75,0.15\right\}$ is tested and compared to baseline prediction.

## 3. Results

**Figure 4:**
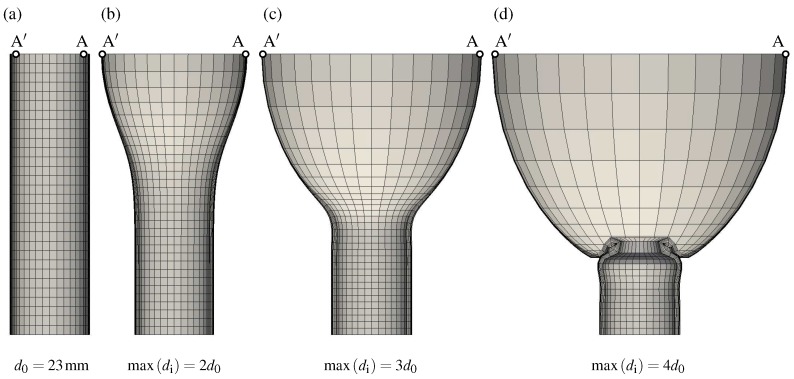
Predicted shapes during Abdominal Aortic Aneurysm (AAA) expansion. (**a**) Normal aorta at homeostasis with inner diameter $d_{0}=23.0$ (mm); Configurations (**b**–**d**) show AAAs at maximum inner diameters of $2d_{0}$, $3d_{0}$, and $4d_{0}$, respectively.

Figure 4 illustrates the predicted AAA shapes, starting with normal aorta at homeostasis (a). At this configuration, the maximum inner diameter is $d_{i}=d_{0}=23$ (mm) and the wall thickness $h_{0}=0.98$ (mm). The maximum inner diameter $d_{i}$ is measured between points $A^{\prime }$ and A shown in Figure 4. Along with the collagen adaptation, the aneurysm diameter and the sac length increased simultaneously over time, see Figure 4b–d. The longitudinal aneurysm growth compressed the non-aneurysmal aorta segment, which finally folded and buckled, see Figure 4d. The simulation was terminated as soon as the finite elements in the folded region became highly distorted.

### 3.1. Study 1: Influence of Collagen Net Growth

**Figure 5:**
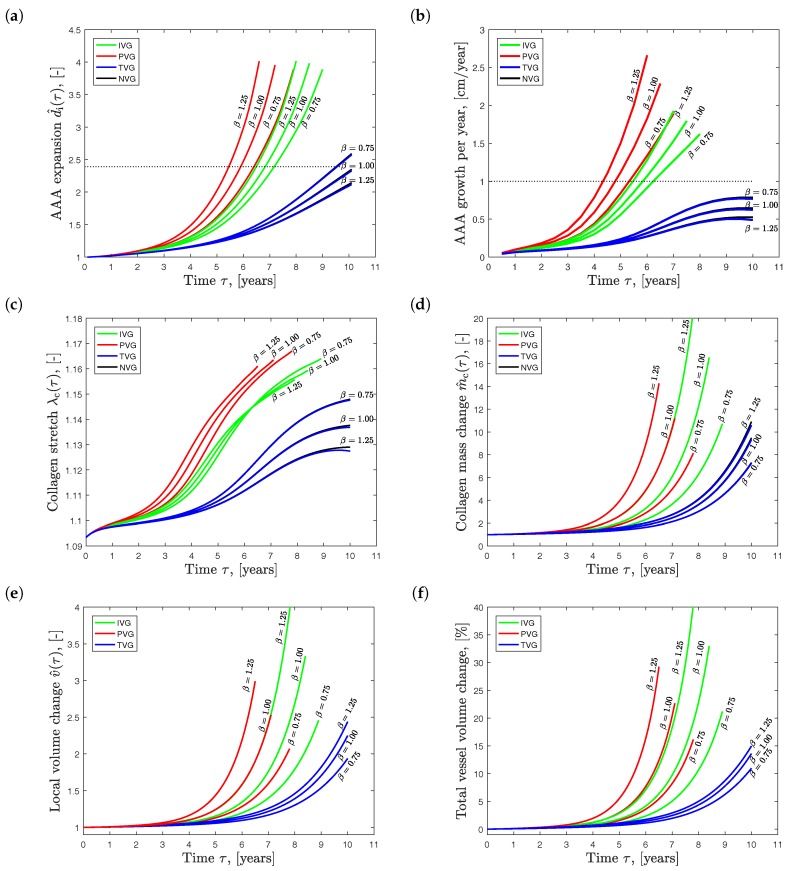
Effect of collagen net growth (parameter β) on the predicted Abdominal Aortic Aneurysm (AAA) expansion over time τ. Isotropic Volume Growth (IVG), in-Plane Volume Growth (PVG), in-Thickness Volume Growth (TVG) and No Volume Growth (NVG) describe growth kinematics. (**a**) AAA expansion ${\hat{d}}_{i}=d_{i}/d_{0}$; (**b**) AAA growth per year; (**c**) collagen stretch $\lambda_{c}$; (**d**) collagen mass change ${\hat{m}}_{c}$; and (**e**) local tissue volume change $\hat{v}$ at the site of the maximum diameter $d_{i}$; (**f**) Total tissue volume change over time. Clinically used AAA repair indication of 55.0 (mm) and 10.0 (mm/year) is shown by the dotted line in (**a**) and (**b**), respectively.

Figure 5 shows the influence of the collagen net growth parameter β on AAA development. The diagrams present quantities at the most expanded location, i.e., at the point A denoted in Figure 4. Figure 5a illustrates the normalized maximum diameter ${\hat{d}}_{i}\left(\tau \right)=d_{i}\left(\tau \right)/d_{0}$, and Figure 5b shows the absolute growth rate, i.e., how much the maximum diameter changes per year. It is noted that both parameters are used for clinical decision making, i.e., indication whether AAA repair should be performed in an individual patient. Elective AAA repair is typically indicated if the maximum diameter is larger than 55.0 (mm) [50], or if it grows faster than 10.0 (mm/year) [51]. These thresholds are denoted by dotted lines in Figure 5a,b.

Predictions following NVG and TVG kinematics show practically the same results. AAA expansion slows down and the aneurysm reaches the same diameter later in time for increasing collagen production, i.e., for increased β parameters. However, IVG and PVG kinematics showed the opposite (and counterintuitive) behavior: AAA expansion accelerated for increasing β parameters.

After five to seven years of growth, predictions based on IVG and PVG kinematics reached AAA repair indications. For TVG and NVG kinematics, only the case β=0.75 (year^−1^) reach AAA repair indication after about 9.5 years.

Figure 5c shows the evolution of the collagen stretch $\lambda_{c}$ over time and at the site of the maximum diameter. For NVG and TVG kinematics, the results were very similar, both quantitatively and qualitatively. An increased β led to less stretched collagen in the wall. In contrast, PVG kinematics showed the counterintuitive result: an increased β caused higher stretched collagen in the vessel wall.

The collagen mass change ${\hat{m}}_{c}$ (see Figure 5d) and the local tissue volume change $\hat{v}$ (see Figure 5e) were consistently higher for increasing values of β, independently from the prescribed growth kinematics. Similar trends are observed in Figure 5f, showing the evolution of the total vessel volume change relative to the volume of the aorta at homeostasis. Despite rather high local volume growth values (up to 300%), the total vessel volume changed only up to 40%.

**Figure 6:**
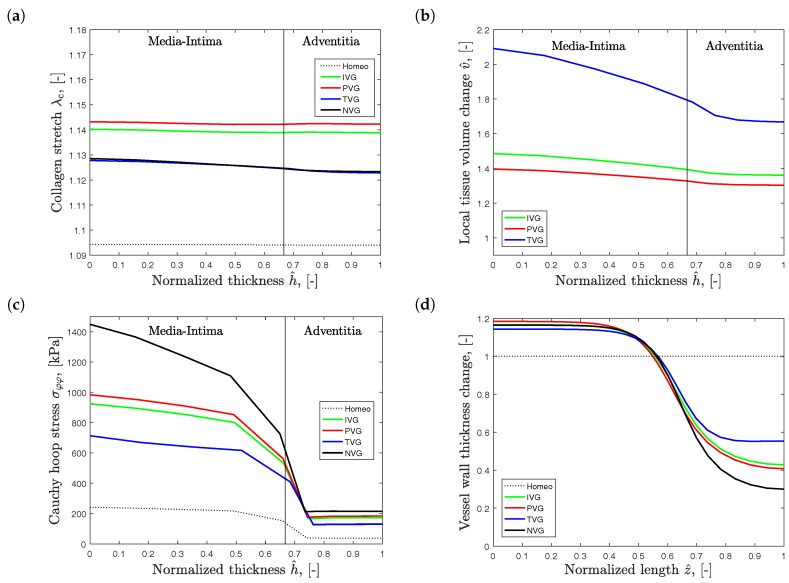
Predicted Abdominal Aortic Aneurysm (AAA) properties at twofold expansion, $d_{i}/d_{0}=2$. Collagen growth is specified by β=1.25 (year^−1^), and Isotropic Volume Growth (IVG), in-Plane Volume Growth (PVG), in-Thickness Volume Growth (TVG) and No Volume Growth (NVG) describe growth kinematics. Transmural plots are shown for (**a**) collagen stretch $\lambda_{c}$; (**b**) local tissue volume change $\hat{v}$; (**c**) Cauchy hoop stress $\sigma_{\varphi \varphi }$; (**d**) Vessel wall thickness, normalized to the thickness $h_{0}$ of aorta at homeostasis, along AAA length.

Figure 6 shows the effect of the different growth models at twofold diameter expansion, observed at the site of the largest diameter. The results have been derived with the collagen net growth parameter of β=1.25 (year^−1^). Figure 6a shows the transmural variation of the collagen stretch $\lambda_{c}$. The stretch values are slightly elevated towards the lumen, and show highest gradients for TVG and NVG kinematics. Collagen stretch is highest for PVG, slightly lower for IVG, and lowest for TVG and NVG.

Figure 6b presents the transmural variation of the normalized tissue volume change $\hat{v}$. Here, an inverted response is observed when compared to the collagen stretch plots. The largest volume change was observed for TVG kinematics, and prescribing PVG resulted in the lowest volume change. In addition, volume change exhibited steeper transmural gradients than the transmural variations of collagen stretch, compare Figure 6a,b.

Finally, Figure 6c presents the transmural changes of the Cauchy hoop stress, and Figure 6d, the variation of vessel wall thickness along the vessel length. Stress and wall thickness are inversely correlated, and highest and lowest stress values are observed for NVG and TVG kinematics, respectively.

### 3.2. Study 2: Influence of Initial Volume Fractions

**Figure 7:**
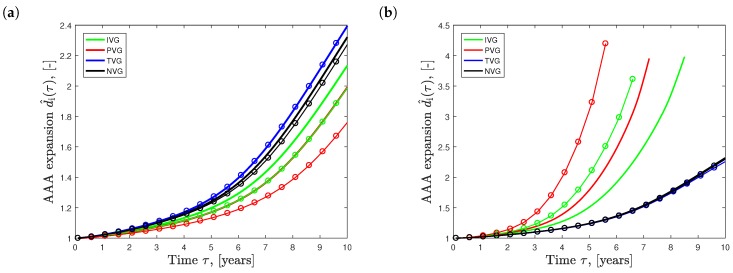
Effect of the initial volume fraction of elastin (**a**) and collagen (**b**) on the evolution of Abdominal Aortic Aneurysm (AAA) diameter ${\hat{d}}_{i}=\left(d_{i}\right)/d_{0}$ over time τ. Isotropic Volume Growth (IVG), in-Plane Volume Growth (IVP), in-Thickness Volume Growth (TVG) and No Volume Growth (NVG) describe growth kinematics. (**a**) AAA expansion with initial elastin volume fractions of $\phi_{e}=0.12$ (solid curves) and $\phi_{e}=0.18$ (solid curves with circles); (**b**) AAA expansion with initial collagen volume fractions of $\phi_{c}^{i}=0.75$ (solid curves) and $\phi_{c}^{i}=0.15$ (solid curves with circles) prescribed in the media.

Figure 7a shows the effect of the different elastin growth (degradation) kinematics upon AAA development in time (solid curves, $\phi_{e}=0.12$). Elastin degradation following TVG kinematics resulted in the fastest AAA expansion, closely followed by NVG kinematics. IVG and PVG kinematics predicted slightly slower aneurysm expansion over time. Similar effects were observed in the tissue patch model shown in Figure 3.

The dashed curves in Figure 7a illustrate AAA expansion at the increased initial elastin volume fraction of $\phi_{e}=0.18$. For PVG, IVG and NVG kinematics, an increased $\phi_{e}$ resulted in slower AAA expansion, with the most pronounced effect seen for PVG. Interestingly, for TVG kinematics, an increased $\phi_{e}$ had no apparent effect on AAA expansion.

Figure 7b shows AAA expansion for initial collagen volume fractions of $\phi_{c}^{i}=\left\{0.75,0.15\right\}$ prescribed in the medial vessel layer. For IVG and PVG kinematics, an increased $\phi_{c}$ led to faster aneurysm growth, while TVG and NVG kinematics showed the opposite. Similar qualitative effects, with significantly lower magnitudes, were observed when changing the initial collagen volume fraction in the adventitia.

## 4. Discussion

The literature reports numerous biomechanical frameworks to model vascular tissue adaptation, each of which uses *ad hoc* assumptions, and much more development is needed before such models can be of any practical use. Relevant experiment data is scarce, such that parameter studies, like the present one, can provide valuable information to guide model development, but may also help to design experiments to explore the response of vascular tissue to mechanical loads.

By prescribing different types of anisotropic volume growth kinematics, we highlighted their severe influence on predicting AAA evolution. Previous membrane implementations of isochoric growth (NVG kinematics) yielded the expected result, i.e., that an increased collagen net production decreased AAA growth, see for example [52]. For TVG kinematics, i.e., where new volume is deposited perpendicular to the tangential plane, our study showed very similar results. Conversely, IVG kinematics (isotropic tissue deposition) and PVG kinematics (tissue deposited in the tangential plane) predicted faster aneurysm expansion at increased collagen net production, a finding that is neither intuitive nor plausible. Therefore, IVG and PVG kinematics should be used with caution when modeling aneurysm growth.

Due to its definition, wall stress is largely influenced by AAA wall thickness. Vessel wall thickness correlates with vessel diameter, which itself depends on factors like age, sex and body size [53]. In addition, the outer border of the adventitia is blurry, and vessel wall thickness measurements are highly influenced by the measurement methods [54]. Most commonly infrarenal aorta wall thickness is reported from in-vivo measurements. A wall thickness of 1.9 (mm) [55] has been reported, which matches data for 70+ subjects [56], i.e., the age group of AAA patients. Assuming 15% circumferential in-vivo deformation, gives a wall thickness of roughly 2.2 (mm) of the stress-free infrarenal aorta wall. In contrast, data from in total 15 in vitro biomechanical studies of AAA wall samples, yielded a median wall thickness of 1.6 (mm), see Table 2 in [57]. These data suggests that aneurysm disease reduces aortic wall thickness on average by 36%. However, AAA wall thickness shows huge inter-patient and intra-patient variabilities, and no statistically significant difference between small and large AAAs was found [58].

At twofold AAA expansion, and with the collagen net growth of β=1.25 (year^−1^), the present study predicted AAA wall thicknesses of 0.28 (mm) and 0.54 (mm) for NVG and TVG kinematics, respectively. Compared to the homeostatic wall thickness of 0.98 (mm) of the normal aorta, this yields a AAA-related wall thickness reductions of 71% and 45%. Hence, even prescribing the extremal case of TVG kinematics, overestimated AAA-related wall-thinning. This discrepancy might be explained by the strict mixture rule of CCD that was applied to collagen. However, the well organized and tightly packed organization of collagen in the normal aorta, might be lost with the course of aneurysm disease. Consequently, collagen might no longer be deposited according to the CCD mixture rules, which in turn requires a thicker wall to host the same amount of collagen. Alternatively, notice that in the simulations, the collagen stretch is above the initial collagen attachment stretch levels (see Figure 5c). If the collagen stretches were lower (for given aneurysm size) then to maintain mechanical equilibrium for decreased collagen stress (given decreased collagen fibre stretch), increases in collagen mass would be required, which again results in an increased wall thickness.

Accurate experimental measurement of individual constituents’ volume fractions is difficult, especially with non-invasive methods. Therefore, it is important to explore the sensitivity of AAA growth models with respect to the initial volume fractions of such individual constituents. Interestingly, NVG and TVG kinematics show very low sensitivity to initial volume fractions of elastin and collagen, such that population-based inputs may be used. In contrast, predictions based on IVG and PVG kinematics are highly sensitive to the initial volume fractions of elastin and collagen.

Once introducing a rupture criterion for the wall, i.e., specifying at which conditions (stress, strain, growth rate, etc.) the aorta wall would rupture, the method applied in this study could in principle be used to predict aneurysm rupture. While the literature reports static strength of the AAA wall, it remains unclear how such strength is influenced by non-physiological collagen turnover, i.e., conditions prior rupture. Consequently, a rupture criterion for the AAA wall would be incomplete in the present context. In addition, technical challenges, such as handling structural instabilities and contact with surrounding organs, would further complicate predicting AAA rupture.

This work used many modeling assumptions, which might be debated. First of all, we used a stretch-based mechanical stimulus with respect to which collagen turnover responds. However, it is still unclear which mechanics-related parameter stimulates collagen turnover, and one could have used a stress-related stimulus instead. The implications of this assumption should be further explored.

Collagen orientations in the normal aorta [31,32,33,34], as well as in the aneurysmatic aorta [35] are dispersed, and the approximation by two families of collagen fibers is rather conceptual. In addition, also the undulation of collagen fibers is dispersed, and collagen fibers gradually engage when loading the vascular wall. Collagen undulation dispersion was not considered in the present work, and the use of a single (deterministic) recruitment stretch aimed at capture salient features of collagen fiber engagement in the vessel wall. More refined models are available [59,60]; their application in the present context might be further explored.

Finally, the presented model does consider neither active stress from SMC, nor the presence of an ILT. While SMC influence the onset and early development of AAAs, the ILT likely effect its later development. Despite challenging, both effects might be considered in a revised AAA growth model.

## 5. Conclusions

This work demonstrated that the choice of tissue growth kinematics is of crucial importance when modeling AAA growth. Specifically, prescribing IVG or PVG kinematics led to highly non-plausible predictions, and models should no longer used such assumptions. Despite TVG kinematics predicted the most reasonable result in this study, it still overestimated wall thinning through aneurysmal progression. However, limited experimental data prevents from a sound validation of modeling assumptions, and much more interdisciplinary experimental work is required before AAA expansion models can be of any practical (clinical) use.

## Figures and Tables

**Table 1:** Reference set of parameters to model Abdominal Aortic Aneurysm (AAA) expansion.

**Reference Geometry and Loading**		**Value**	
Inner radius	$R_{i}$	8.4 (mm)	
Artery length	*L*	147.4 (mm)	
Media-intima thickness	$H_{M}$	1.18 (mm)	
Adventitia thickness	$H_{A}$	0.59 (mm)	
Inner pressure	$p_{i}$	16 (kPa)	
Axial pre-stretch	$\lambda_{z}$	1.2	
**Material Model**		**Media**	**Adventitia**
Elastin shear modulus	$\mu_{e}$	133.81 (kPa)	0 (kPa)
Elastin initial volume fraction	$\phi_{e}$	0.12	0
Ground matrix shear modulus	$\mu_{g}$	33.45 (kPa)	33.45 (kPa)
Ground matrix initial volume fraction	$\phi_{g}$	0.73	0.86
Collagen parameter	$k_{c1}$	3.52 (kPa)	3.52 (kPa)
Collagen parameter	$k_{c2}$	40	40
Collagen family initial volume fraction	$\phi_{c}^{i}$	0.075	0.075
Fictitious bulk modulus	κ	100 (kPa)	100 (kPa)
**Aneurysm growth**		Equation	Value
Collagen remodeling rate	α	Equation (18)${}_{1}$	0.6 (year^−1^)
Collagen net growth rate	β	Equation (18)${}_{2}$	1.0 (year^−1^)
Collagen attachment stretch	$\lambda_{a}$	Equation (17)	1.093
Initial value collagen recruitment stretch	$\lambda_{\mathrm{rec}}$	Equation (18)${}_{1}$	1.13
Target amount of elastin	$c_{\min}$	Equation (19)	0.6
Degradation time	*T*	Equation (19)	10 (years)
Shape parameter	$m_{1}$	Equation (19)	20
